# Emotional intelligence as a factor in the efficient self-regulation of functional states under work strain

**DOI:** 10.1192/j.eurpsy.2021.1234

**Published:** 2021-08-13

**Authors:** T. Zlokazova, A. Kuznetsova

**Affiliations:** Faculty Of Psychology, Lomonosov Moscow state University, Moscow, Russian Federation

**Keywords:** Emotional intelligence, Work stress, self-regulation, work competence

## Abstract

**Introduction:**

Self-regulation of emotions is viewed as one of the key skills in various socionomic professions (Kuznetsova & Titova, 2016; Spencer & Spencer, 2008), including psychology. Emotional intelligence (EI) can be seen as a vital competence for counseling psychologists, as well as a factor in an efficient human functional state’s (HFS) self-regulation under stressful work conditions.

**Objectives:**

This study aims to assess the EI level in psychologists involved in a long-term relief programme for people affected by an industrial accident, and to reveal interrelations between EI and the efficiency of acquisition of new self-regulation skills.

**Methods:**

The training course was designed for psychologists (n=15) in order to develop new stress-management counseling skills. It included: progressive relaxation, ideomotor and visualization exercises; autogenic formulae. Data were obtained from HFS evaluation questionnaires (Leonova, Zlokazova, Kachina & Kuznetsova, 2013), and the EI inventory (Manoylova, 2004).

**Results:**

The mean EI level was high among the psychologists, although there were variations. Data confirmed rapid progress in the acquisition of self-regulation skills, and demonstrated a prolonged effect of stress (p=0,052) and fatigue (p<0,001) reduction (Friedman’s test). The EI level showed correlations (Spearman’s test) with HFS measures: by the end of the course psychologists with a higher EI reported higher psychological comfort (p<0,05) and lower acute fatigue (p<0,01) after relaxation exercises.

**Conclusions:**

Although the sample size was small, the data, obtained from described above unique group of counseling psychologists, helped reveal the link between EI and efficient HFS self-regulation.

